# Chronic chlorothalonil exposure inhibits locomotion and interferes with the gut-liver axis in *Pelophylax nigromaculatus* tadpoles

**DOI:** 10.1038/s41598-025-98081-1

**Published:** 2025-04-25

**Authors:** Minyi Huang, Yuhao Zhang, Xiang Xu, Renyan Duan, Hui Yang

**Affiliations:** 1 College of Agriculture and Biotechnology, Hunan University of Humanities, Science and Technology, Loudi, 417000 Hunan China; 2 Key Laboratory of Development, Utilization, Quality and Safety Control of Characteristic Agricultural Resources in Central Hunan Province, Loudi, 417000 Hunan China

**Keywords:** Chlorothalonil, Locomotion, Gut-liver axis, Environmental sciences, Metabolomics

## Abstract

Chlorothalonil is a widely used fungicide that has a negative effect on individual movement, but its impact pathway needs further refinement. Here, the effects of exposure to chlorothalonil on the locomotion behavior of *Pelophylax nigromaculatus* tadpoles (GS23) were measured at three different levels (0 µg/L, 10 µg/L, and 50 µg/L), and the possible pathways of its effects were analyzed from the gut-liver axis. Chlorothalonil exposure levels of 10 µg/L and 50 µg/L significantly reduced the average speed of *P. nigromaculatus* tadpoles by 26% and 32.7%, respectively, and significantly decreased the locomotor frequency by 27.1% and 58.6%, respectively. Gut microbiota analysis revealed chlorothalonil exposure significantly increased the abundance of *Firmicutes*, while significantly decreased the abundance of *Actinobacteriota*, *Pseudomonas*, and *Rhodococcus*. Metabolomics analysis identified that chlorothalonil treatment changed amino acid-related metabolism pathways in the gut and liver and altered the glycerophospholipid metabolism pathway in the liver. This study indicated that chlorothalonil can affect individual locomotor abilities and interfering with the gut-liver axis of aquatic animals. These findings establish that chlorothalonil compromises aquatic organism motility through a multi-target mechanism involving gut microbiota modulation, amino acid metabolic interference, and hepatic lipid pathway disruption.

**Supplementary Information:**

The online version contains supplementary material available at 10.1038/s41598-025-98081-1.

## Introduction

Locomotion serves as a critical survival mechanism for animals, enabling essential ecological functions including foraging, predator avoidance, and reproductive success^1–3^. As a sensitive biomarker of environmental toxicity, locomotor capacity has been widely adopted to evaluate pesticide impacts on aquatic organismsn^4,5^. Recent toxicological studies demonstrates that pesticide exposure induces multifaceted locomotor impairments in amphibians including reduced motility, movement restriction, and completely immobile^6–8^. These studies confirm the negative effects of pesticides on animal movement, but their pathways of influence need further refinement.

The gut and liver of the gut-liver axis are two important components of the vertebrate digestive system, which has an important role in energy metabolism and affects the locomotor state^9,10^. The gut-liver axis relies on normal liver function, an intact intestinal barrier, and a healthy intestinal microbiota environment to carry out its biological functions^11^. Pesticides have been shown to affect intestinal and liver homeostasis in animals^12^. Pesticide exposure can damage the gut microbiota and gut metabolites, affecting individual swimming and social behavior^13,14^. In addition, exposure to pesticides can affect liver glutathione S-transferase and carboxylesterase activity^15^, disrupt TCA cycle and amino acid metabolism related to liver energy production, and reduce individual swimming distance and average speed^16^. The study of the gut-liver axis in toxicological studies holds great promise for a deeper understanding of the toxic effects and mechanisms of pesticides^17^. However, these studies have mainly focused on the effects of pesticides on a single organ of the gut-liver axis, with fewer studies considering the interaction of the gut and liver. It is necessary to explore the effects of pesticides on animals from the point of view of the entire gut-liver axis.

The pesticide chlorothalonil is widely used in agriculture as a broad-spectrum, non-systemic fungicide for the control of foliar fungal diseases in fruits, vegetables, and ornamentals^18^. In the US, chlorothalonil is mainly used on tomatoes, peanuts, and golf courses, accounting for about 15% of total fungicide use and exceeds 5 × 10^6^ kg per year^19^. In China, the annual production of chlorothalonil is more than 8 × 10^6^ kg^20^. Chlorothalonil is commonly detected in surface and groundwater^21^. The concentration of chlorothalonil detectable in water ranges from 0.008 µg/L to 29.78 µg/L^22^. A research report found that the concentration in runoff could reach up to 500 µg/L immediately after the use of chlorothalonil, and that two days after use the concentration of chlorothalonil in runoff ranged from 50 to 130 µg/L^19^. There is considerable evidence that chlorothalonil is highly toxic to aquatic organisms^19,23^.

Amphibians are both aquatic and terrestrial animals with high skin permeability and are vulnerable to pesticides in the water column^24,25^. Tadpoles are a commonly used model for the study of locomotor changes following pesticide exposure^26–28^. Although these studies have reported the effects of exposure to chlorothalonil on amphibians, research on the toxicity of chlorothalonil to amphibians is still limited. *Pelophylax nigromaculatus* is a widely distributed amphibian in China, Korea, and Japan. It plays an important role in agroecosystems, protecting crops and predating pests. Furthermore, it can serve as an important animal model for evaluating the ecotoxicological effects of environmental pollution^29,30^.

This study aimed to investigate how chlorothalonil exposure affects the gut-liver axis and locomotion in *P. nigromaculatus* tadpoles. Here, we have set two processing concentrations (10 µg/L and 50 µg/L). 10 µg/L is the environmental concentration of most water bodies, while 50 µg/L is the detectable concentration in some agricultural water bodies^19,22^. We hypothesized that exposure to chlorothalonil may not only interfere with locomotion, but also alter the gut microbial composition, gut metabolism, and liver metabolism in *P. nigromaculatus* tadpoles. To test this hypothesis, *P. nigromaculatus* tadpoles were exposed to chlorothalonil at three different concentrations (0 µg/L, 10 µg/L and 50 µg/L) for 30 days. Subsequently, alterations in locomotion, gut microbial composition, gut metabolism, and liver metabolism were examined. This study provides a reference point for considering chlorothalonil use in future amphibian conservation efforts.

## Materials and methods

### Experimental materials

Chlorothalonil (CAS No: 1897456, purity ≥ 98%) and its organic solvent dimethyl sulfoxide (DMSO) (CAS No: 67-68-5) were obtained from Shanghai Aladdin Reagent Co. The fertilized eggs of *P. nigromaculatus* were procured from a breeding site in Loudi City, Hunan Province, China. The incubation process was conducted following the methodologies and environmental conditions outlined by Lou et al.^31^. A total of 360 healthy GS23 frog tadpoles with similar body weight and length were selected and randomly divided into three treatments: a control group (CON), a 10 µg/L chlorothalonil treatment (CT10), and a 50 µg/L chlorothalonil treatment (CT50). Chlorothalonil is slightly soluble in water and easily soluble in organic solvents. In toxicity experiments, DMSO is commonly used as a co-solvent for chlorothalonil to improve its solubility^19^. The concentration of DMSO in the control group (< 0.05%) was suitable for carrying out the experiments^32,33^. The configured concentrations were validated using high-performance liquid chromatography, with validated concentrations of 9.76 ± 0.84 µg/L or 48.63 ± 3.25 µg/L, respectively.

Each group was comprised of 120 tadpoles, with three replicates of 40 tadpoles per group. *P. nigromaculatus* tadpoles were reared at a temperature of 23.2 °C ± 1.5 °C, dissolved oxygen of 7.3–8.2 mg/L, pH of 6.4–6.9 and 14 h/10 h (light/dark) environmental conditions. *P. nigromaculatus* tadpoles from each group were reared in tanks (50 cm × 35 cm × 25 cm) containing 4 L of aerated water, with a treatment solution added to each tank. Cooked lettuce leaves were fed once a day and the treatment solution was renewed once a day^34^.

### Locomotor behavior analysis

The locomotor behavior of *P. nigromaculatus* tadpoles was analyzed after 30 days of chlorothalonil exposure (GS23-GS36) and 15 *P. nigromaculatus* tadpoles were randomly selected from the three replicates of each treatment group. *P. nigromaculatus* tadpoles were brought into the isolated behavioral room one hour prior to the behavioral test to acclimatize to the new environment, and then behavioral trials were conducted between 10:00 and 13:00. Randomly selected tadpoles were placed in a circular water tank with a diameter of 30 cm and a height of 6 cm for filming. The filming time for each tadpole’s movement behavior was 10 min. During the filming process, a Sony color camera recorded at a speed of 6 frames per second. To ensure accuracy, our filming process was manually confirmed by two observers who calibrated the video tracking and recognition accuracy of *P. nigromaculatus* tadpoles. After filming, the video was analyzed using Noldus’ EthoVision XT 15 software to obtain locomotor behavior data^35,36^.

### Sampling

After behavioral analysis, all *P. nigromaculatus* tadpoles from the control and each treatment group were euthanized in 1% tricaine methanesulfonate (Sigma Aldrich, CAS 886-86-2). Gut contents, gut tissue, and liver tissue were extracted using the sampling method of Huang et al.^30^ Gut contents were used to detect gut microorganisms and gut metabolites. Liver tissues were used to detect liver metabolites. All experiments were carried out in accordance with relevant guidelines and regulations.

### Ethical approval and informed consent

The present study was approved by the Hunan University of Humanities and Technology, China (202205). Meanwhile, the ARRIVE guidelines (https://arriveguidelines.org) were strictly followed in this study.

### Analysis of gut microorganisms

Total DNA of the intestinal flora was extracted from samples of the intestinal contents of *P. nigromaculatus* tadpoles. PCR amplification of the 16 S rRNA gene was performed using forward primer (338 F) and reverse primer (806R). The products were purified and analyzed on a 2% agarose gel. Sequencing was performed on Illumina’s Miseq PE300/NovaSeq PE250 platform^34^. Fastp software was used for quality control of the raw sequences. Splicing was performed with FLASH software. Sequences were OTU clustered and chimeras were removed based on 97% similarity with the UPARSE software. Species classification of each sequence was annotated with the RDP classifier.

### Metabolomics analysis

Gut contents or liver tissues were subjected to non-targeted metabolomics analyses following the method of Huang et al.^34^ In brief, the intestinal contents or liver tissue of *P. nigromaculatus* tadpoles were mixed and pre-treated. Samples were centrifuged and analysed by mass spectrometry. The mass spectrometry analysis was performed under the following conditions: the ion source temperature (425 °C), the collision energy gradient (20–60 V), the spray voltage (positive ion mode: 3500 V and negative ion mode: 3500 V), full MS resolution (60000), and the mass-to-charge (m/z) scan range(70-1050)^37^. The raw LC/MS data were imported into Progenesis QI 2.3 software (Waters Corporation) and converted into a three-dimensional data matrix (CSV format), comprising sample identifiers, annotated metabolite features, and spectral intensity values. Metabolic features with > 20% missing values within any experimental group was removed. Each metabolite was normalized by summation. Metabolites with a relative standard deviation (RSD) < 30% in the quality control samples were retained to ensure analytical reproducibility. The raw data were transformed and corrected by log10 to reduce heteroscedasticity and stabilize variance, enhancing the reliability of subsequent multivariate analyses.

### Statistical analyses

The alpha diversity indices, including the Inverse-Simpson index, the Pielou index, the Shannon’s evenness index, and the Berger-Parker index, were calculated by QIIME2 software. Species diversity could be represented by the Inverse-Simpson index and the Berger-Parker index^38,39^. The Pielou index and Shannon’s evenness index were used to assess community evenness^40,41^. Beta diversity analysis and sample grouping analysis were performed separately. A one-way ANOVA was used to determine the significance of locomotive behavior, intestinal microbiota, and liver and gut differential metabolites between treatment groups. Sample comparisons and KEGG topology analysis were performed for the gut and liver metabolomes using Partial Least Squares Discriminant Analysis (PLS-DA) and relative-betweenness centrality calculations, respectively^42–44^. The criterion for pathway screening in the KEGG topology analysis of the gut metabolome was an impact value > 0.15. The screening criteria for pathways in the KEGG topology map of the liver metabolome was an impact value > 0.1. Spearman correlation analysis was used to assess the correlation between liver metabolites and intestinal microbiota.

## Results

### Effects of Chlorothalonil on locomotor behavior of *P. nigromaculatus* tadpoles

**Fig. 1:**
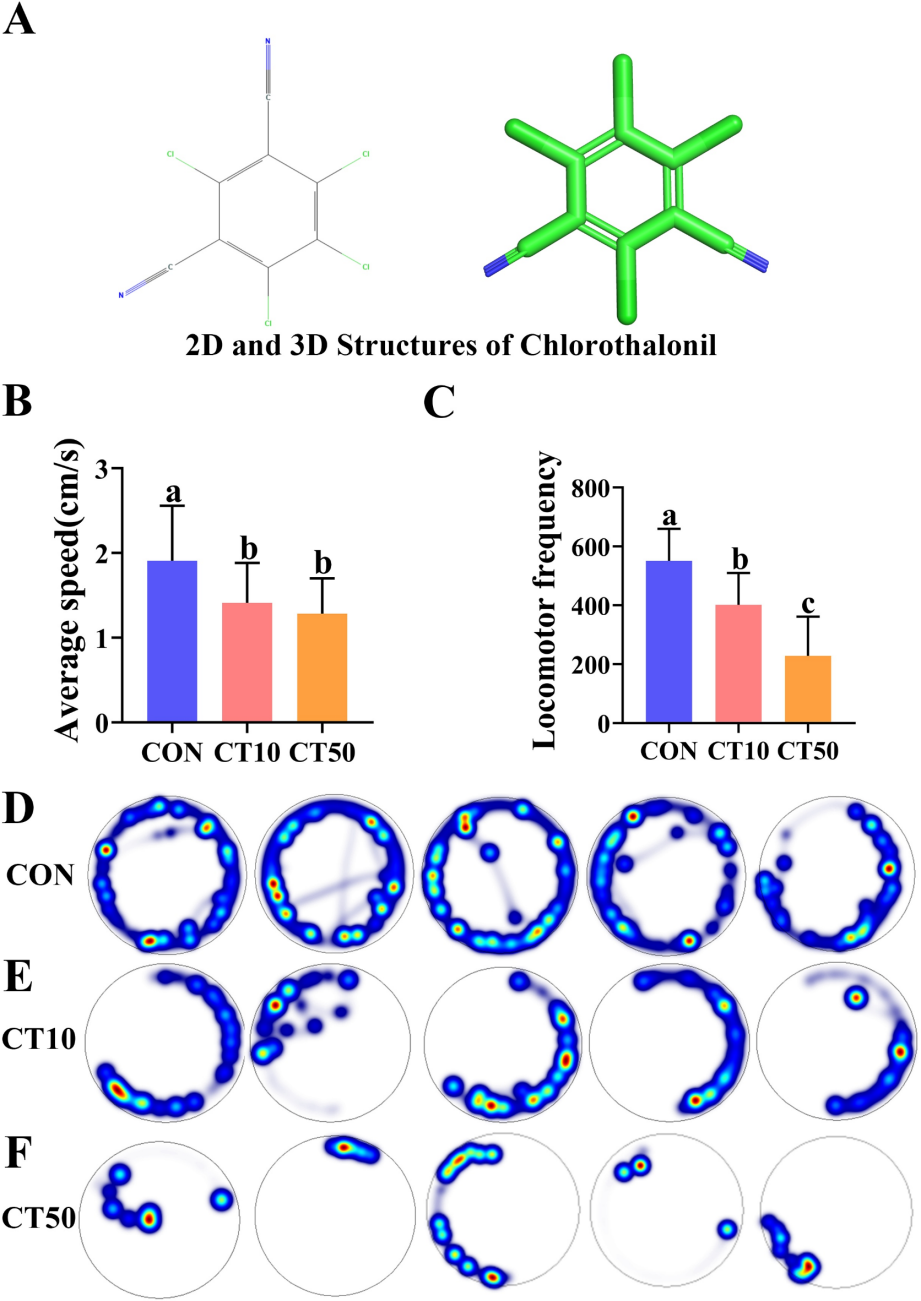
Chlorothalonil structures and locomotions of *P. nigromaculatus* tadpoles among the CON, CT10 group and CT50 group. (A) 2d and 3d structures of chlorothalonil. (B, C) Average speed and locomotor frequency. Different letters among the three groups indicate significant differences. (D–F) Heatmaps of behavioural trajectories of locomotions in the CON, CT10 group and CT50 group, respectively. Blue and green = low frequency, yellow and red = high frequency.

Figure 1A showed the 2d and 3d structures of chlorothalonil. Behavioral differences between CON, CT10 and CT50 were compared, including the average speed and frequency of movement. CT10 significantly reduced the average speed (*P* = 0.013) and locomotor frequency (*P* = 0.001) of *P. nigromaculatus* tadpoles by approximately 26% and 27.1%, respectively, compared to CON. CT50 also significantly reduced the average speed (*P* = 0.002) and locomotor frequency (*P* = 0.26 × 10^−8^) of *P. nigromaculatus* tadpoles by approximately 32.7% and 58.6%, respectively (Fig. 1B, C). Both CT10 and CT50 significantly inhibited the locomotor activity of *P. nigromaculatus* tadpoles (Fig. 1D–F). Overall, CT50 inhibited the locomotion of *P. nigromaculatus* tadpoles more than CT10 (Fig. 1).

### Effects of Chlorothalonil on intestinal microbiota in *P. nigromaculatus* tadpoles

**Fig. 2:**
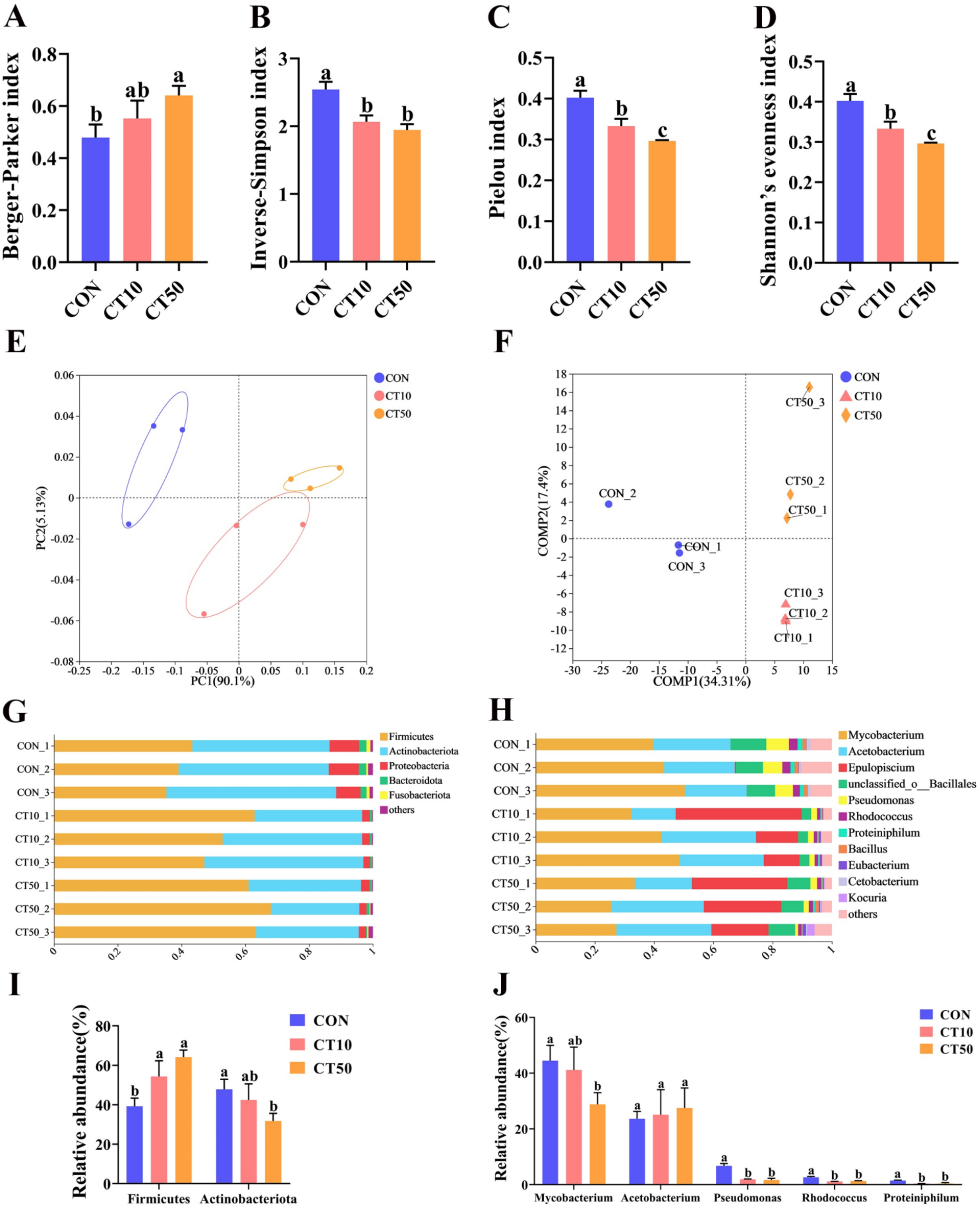
Effect of CON, CT10 and CT50 on gut microorganism in *P. nigromaculatus* tadpoles. (A) Berger-Parker index, (B) Inverse-Simpson index, (C) Pielou index, (D) Shannon’s evenness index. (E) Principal Coordinates Analysis (PCoA) base on Bray-Curtis distance. (F) Partial Least Squares Discriminant Analysis (PLS-DA). PCoA is a non-constrained method of data dimensionality reduction analysis, and PCoA is not constrained by distance algorithms, similar to PCA analysis. PLS-DA analysis, a multivariate statistical analysis method used for discriminant analysis, determines how a research subject is classified based on the values of a number of variables that are observed or measured. Average relative abundances of the gut microorganism at phylum level (G) and genus level (H). The differences between the three groups at the phylum level (I) and genus level (J). Different letters indicate significant differences.

The sparse curve showed that sampling coverage was high (Fig. S1A). CON, CT10, and CT50 had 83, 4, and 47 unique OTUs, respectively, and 202 shared OTUs (Fig. S1B). Compared to CON, CT10 significantly decreased the Inverse-Simpson index (*P* = 0.001), Pielou index (*P* = 0.001), and Shannon’s evenness index (*P* = 0.001) by 18.8%, 17.2%, and 17.2%, respectively (Fig. 2B–D). Compared to CON, CT50 significantly decreased the Inverse-Simpson index (*P* = 0.29 × 10^–3^), Pielou index (*P* = 0.96 × 10^–4^), and Shannon’s evenness index (*P* = 0.96 × 10^–4^) by 23.5%, 26.3%, and 26.3%, respectively, while CT50 significantly increased the Berger-Parker index (*P* = 0.96 × 10^–2^) by 33.9% (Fig. 2A–D). Moreover, compared to CT10, CT50 significantly decreased the Pielou index (*P* = 0.019) and Shannon’s evenness index (*P* = 0.96 × 10^−4^) by 11.1% and 11.0%, respectively (Fig. 2D). The principal coordinate analysis (PCoA) and PLS-DA analyses showed that the gut microbial samples from these three treatments (CON, CT10, and CT50) were classified into three distinct groups (Fig. 2E, F).

At the phylum level, *Firmicutes* (35.2–68.1%), *Actinobacteriota* (27.7–53.3%), and *Proteobacteria* (2.1–9.5%) were dominant (Fig. 2G). At the genus level, some important bacterial genera were found, such as *Mycobacterium*, *Pseudomonas*, *Rhodococcus*, and *Proteiniphilum* (Fig. 2H). Compared to CON, CT10 and CT50 significantly increased the abundance of *Firmicutes* (*p* = 0.016) and *Firmicute*s (*p* = 0.002) by 38.5% and 63.4%, respectively, while CT50 significantly decreased the abundance of *Actinobacteriota* (*p* = 0.017) by 33.6% (Fig. 2I). Compared to CON, CT10 significantly reduced the abundance of *Pseudomonas* (*p* = 0.65 × 10^−4^), *Rhodococcus* (*p* = 0.54 × 10^−4^), and *Proteiniphilum* (*p* = 0.001) by 71.5%, 57.9% and 74.0%, respectively (Fig. 2J), while CT50 significantly reduced the abundance of *Mycobacterium* (*p* = 0.021), *Pseudomonas* (*p* = 0.49 × 10^−4^), *Rhodococcus* (*p* = 0.11 × 10^−3^), and *Proteiniphilum* (*p* = 0.001) by 35.2%, 75.2%, 51.1% and 70.8%, respectively (Fig. 2J).

### Effects of chlorothalonil on the gut metabolome of *P. nigromaculatus* tadpoles

**Fig. 3:**
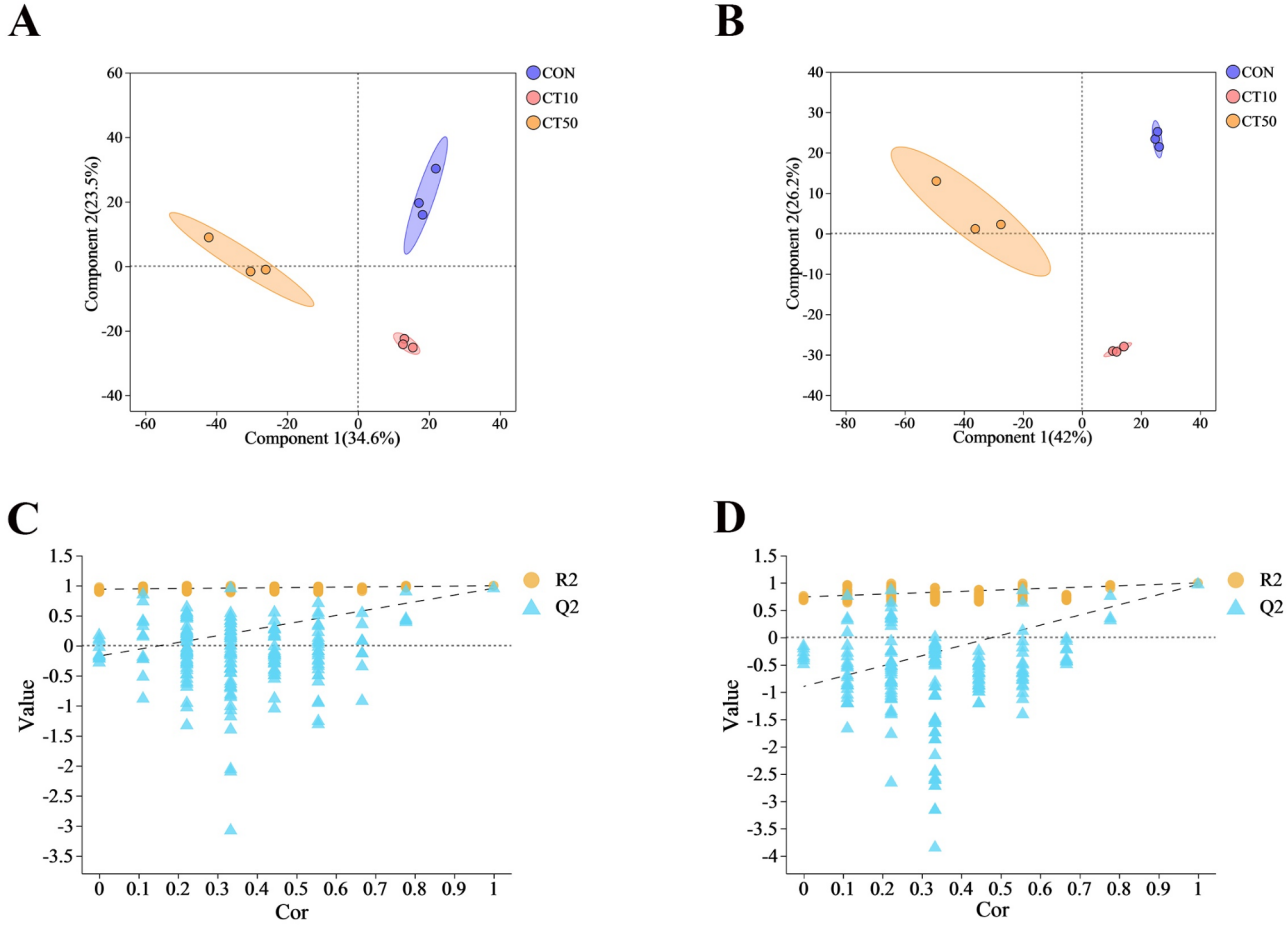
Non-targeted metabolomics analysis of gut of *P. nigromaculatus* tadpoles. PLS-DA score plots from the CON, CT10 and CT50 in (A) positive mode and (B) negative mode. (C) and (D) PLS-DA model validation. Validation plots were obtained in (A) positive mode (R2 = 0.937, Q2 = − 0.1748) and (B) negative mode (R2 = 0.742, Q2 = − 0.8969).

**Fig. 4:**
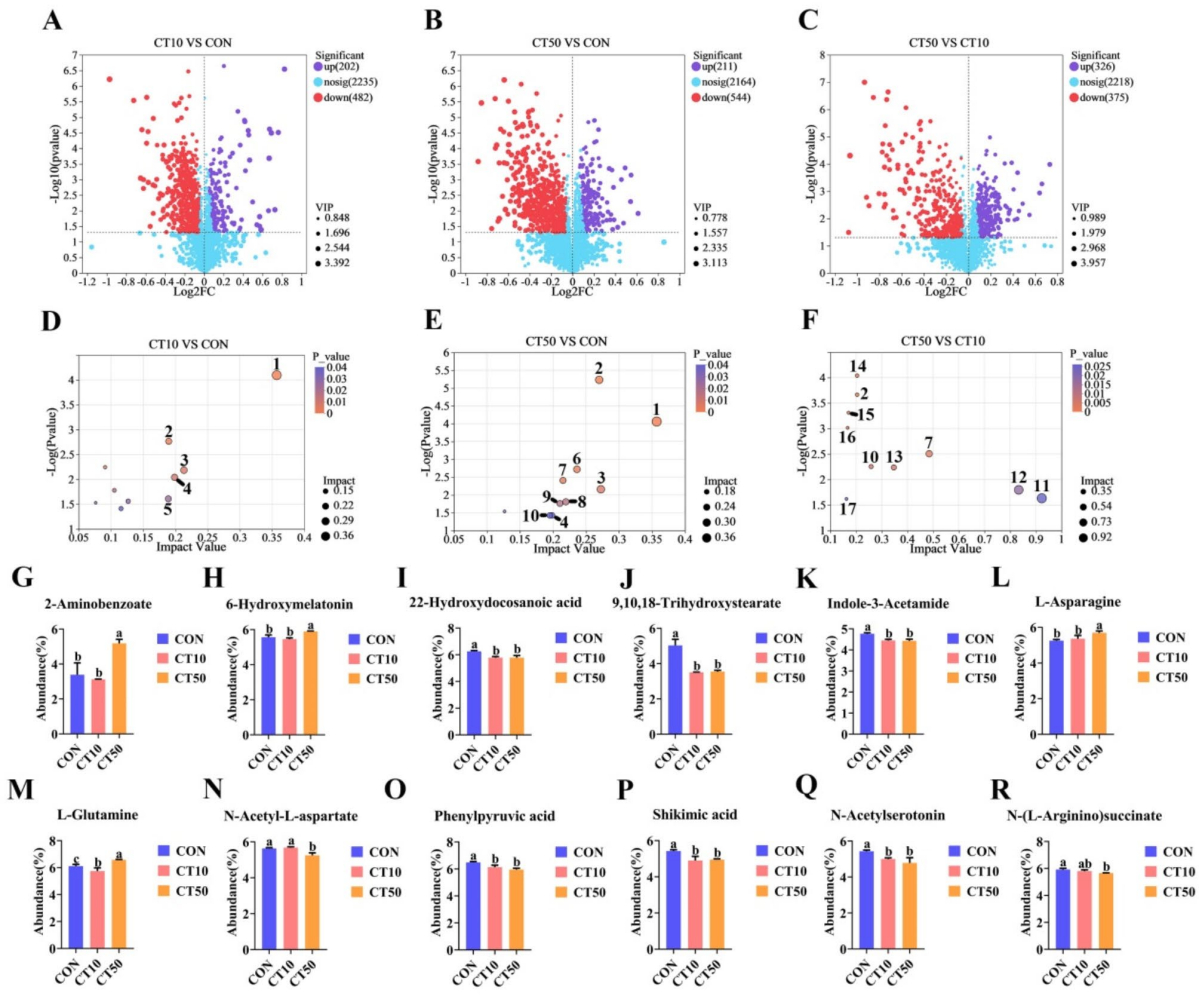
Effect of CON, CT10 and CT50 on gut metabolome of *P. nigromaculatus* tadpoles. Different letters indicated significant differences. (1) Cutin, suberine and wax biosynthesis, (2) Tryptophan metabolism, (3) Phenylalanine, tyrosine and tryptophan biosynthesis, (4) Folate biosynthesis, (5) Nicotinate and nicotinamide metabolism, (6) Arginine biosynthesis, (7) Alanine, aspartate and glutamate metabolism, (8) Biotin metabolism, (9) Tropane, piperidine and pyridine alkaloid biosynthesis, (10) Histidine metabolism, (11) Betalain biosynthesis, (12) One carbon pool by folate, (13) Ribotfavin metabolism, (14) Lysine biosynthesis, (15) Lysine degradation, (16) Arginine and proline metabolism, (17) Glycerophospholipid metabolism.

The PLS-DA model showed that there was a significant separation between CON, CT10, and CT50 (Fig. 3A, B). The groups were well classified, and the validation plots of the PLS-DA showed that the PLS-DA model was reliable (Fig. 3C, D). Compared to CON, CT10 significantly increased the abundance of 202 metabolites and significantly decreased the abundance of 482 metabolites in the gut, while CT50 significantly increased the abundance of 211 metabolites and significantly decreased the abundance of 544 metabolites (Fig. 4A, B). Compared to CT10, CT50 showed a significant increase in 326 metabolites and a significant decrease in 375 metabolites (Fig. 4C).

KEGG topology analysis revealed that the main metabolic pathways that were different between CT10 and CON were (1) cutin, suberine and wax biosynthesis, (2) tryptophan metabolism, and (3) phenylalanine, tyrosine and tryptophan biosynthesis (Fig. 4D). The main metabolic pathways that were different between CT50 group and CON were (1) cutin, suberine and wax biosynthesis, (2) tryptophan metabolism, (3) phenylalanine, tyrosine and tryptophan biosynthesis (Fig. 4E). The main metabolic pathways that were different between CT50 and CT10 were (1) histidine metabolism, (2) alanine, aspartate and glutamate metabolism, and (3) glycerophospholipid metabolism (Fig. 4F).

Compared to CON, CT10 significantly decreased the abundance of 22-hydroxydocosanoic acid (*P* = 0.002) (about 7.6%) (Fig. 4I), 9, 10, 18-trihydroxystearate (*P* = 0.91 × 10^−4^) (about 30.5%) (Fig. 4J), indole-3-acetamide (*P* = 0.001) (about 6.6%) (Fig. 4K), L-glutamine (*P* = 0. 03) (about 6.0%) (Fig. 4M), N-acetylserotonin (*P* = 0.025) (about 8.7%) (Fig. 4O), phenylpyruvic acid (*P* = 0.007) (about 5.4%) (Fig. 4P) and shikimic acid (*P* = 0.004) (about 9.7%) (Fig. 4Q).

Compared to CON, CT50 significantly increased the abundance of 2-aminobenzoate (*P* = 0.002) (about 53%), 6-hydroxymelatonin (*P* = 0.004) (about 6%), L-asparagine (*P* = 0.006) (about 8.4%) and L-glutamine (*P* = 0.011) (about 7%), while CT50 significantly decreased the abundance of 22-hydroxydocosanoic acid (*P* = 0.002) (about 7.6%), indole-3-acetamide (*P* = 0.001) (about 7%), N-acetyl-L-aspartate (*P* = 0.002) (about 6.8%), phenylpyruvic acid (*P* = 0.001) (about 8.2%), shikimic acid (*P* = 0.006) (about 8.8%), N-acetylserotonin (*P* = 0.004) (about 12%), 9, 10, 18-Trihydroxystearate (*P* = 0.107 × 10^−3^) (about 29.6%) and N-(L-arginino) succinate (*P* = 0.005) (about 4.5%) (Fig. 4G–R).

In addition, compared to CT10, CT50 significantly decreased the abundance of N-acetyl-L-aspartate (*P* = 0.001) (about 7.5%), while CT50 significantly increased the abundance of 2-aminobenzoate (*P* = 0.001) (about 66%), 6-hydroxymelatonin (*P* = 0.001) (about 8.1%), L-asparagine (*P* = 0.019) (about 6.2%) and L-glutamine (*P* = 0.001) (about 14.5%) (Fig. 4G, H, L, M, N).

### Effects of chlorothalonil on the liver metabolome of *P. nigromaculatus* tadpoles

**Fig. 5:**
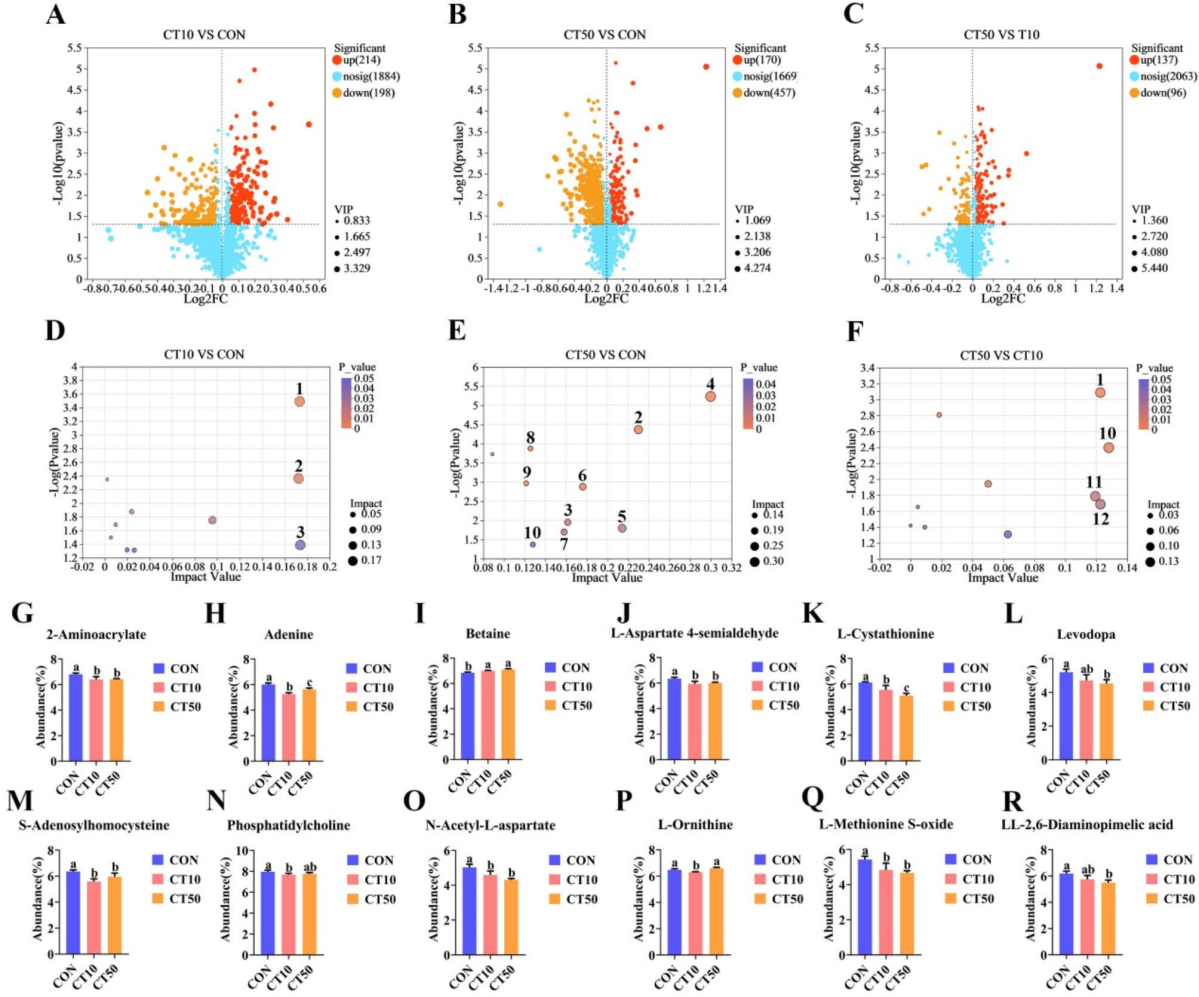
Effect of CON, CT10 and CT50 on liver metabolome of *P. nigromaculatus* tadpoles. Different letters indicated significant differences. (1) Glycerophospholipid metabolism, (2) Cysteine and methionine metabolism, (3) Pantothenate and CoA biosynthesis, (4) Tyrosine metabolism, (5) Clycolysis, (6) Alanine, aspartate and glutamate metabolism, (7) Lysine biosynthesis, (8) Glycine, serine and threonine metabolism, (9) Lysine degradation, (10) Purine metabolism, (11) Arginine biosynthesis, (12) Arginine and proline metabolism. PC(18:3(9Z,12Z,15Z)/18:3(6Z,9Z,12Z)) (Phosphatidylcholine).

The PLS-DA model found a significant separation between CON, CT10, and CT50 (Fig. S2A, B). The groups were well classified, and the validation plots of PLS-DA also showed that the PLS-DA model was reliable (Fig. S2C, D). Compared to CON, CT10 and CT50 significantly increased the abundance of 214 and 170 metabolites in the liver, respectively, and significantly decreased the abundance of 198 and 457 metabolites, respectively. Compared to CT10, CT50 significantly increased the abundance of 137 metabolites, while CT50 significantly decreased the abundance of 96 metabolites (Fig. 5A–C).

KEGG topology analysis revealed that the main metabolic pathways with differences between CT10 group and CON were (1) glycerophospholipid metabolism, (2) cysteine and methionine metabolism, and (3) pantothenate and CoA biosynthesis (Fig. 5D). The main metabolic pathways with differences between CT50 group and CON were (1) tyrosine metabolism, (2) cysteine and methionine metabolism, and (3) alanine, aspartate and glutamate metabolism (Fig. 5E). The main metabolic pathways with differences between CT50 group and CT10 group were (1) glycerophospholipid metabolism, (2) purine metabolism, (3) arginine biosynthesis, and (4) arginine and proline metabolism (Fig. 5F).

Compared to CON, CT10 significantly decreased the abundance of 2-aminoacrylate (*P* = 0.015) (about 6%), adenine (*P* = 0.213 × 10^−3^) (about 12.6%), L-aspartate 4-semialdehyde (*P* = 0.01) (about 6.5%e), L-cystathionine (*P* = 0.021) (about 9.3%), S-adenosylhomoeysteine (*P* = 0.003) (about 12.2%), phosphatidylcholine (*P* = 0.019) (about 3.5%), L-methionine S-oxide (*P* = 0.031) (about 10.9%), L-oraithine (*P* = 0.024) (about 2.7% ), and N-acetyl-L-aspartate (*P* = 0.021) (about 8.7%), while CT10 significantly increased the abundance of betaine (*P* = 0.027) (about 2.1%) (Fig. 5G-Q). Compared to CON, CT50 significantly decreased the abundance of 2-aminoacrylate (*P* = 0.018) (about 5.7%), adenine (*P* = 0.007) (about 6.4%), L-aspartate 4-semialdehyde (*P* = 0.02) (about 5.4%), L-cystathionine (*P* = 0.001) (about 16.9%), levodopa (*P* = 0.019) (about 13.0%), N-acetyl-L-aspartate (*P* = 0.002) (about 14.1%), L-methionine S-oxide (*P* = 0.011) (about 14.0%), S-adenosylhomoeysteine (*P* = 0.024) (about 6.4%) and LL-2,6-diaminopimelic acid (*P* = 0.011) (about 11.1%), while CT50 significantly increased the abundance of betaine (*P* = 0.002) (about 3.6%) (Fig. 5G–R). In addition, compared to CT10, CT50 significantly increased the abundance of adenine (*P* = 0.008) (about 7.0%) and L-oraithine (*P* = 0.003) (about 4.5%), whereas CT50 significantly decreased the abundance of L-cystathionine (*P* = 0.045) (about 8.3%) (Fig. 5H, K, P).

### Correlation analysis between intestinal microbiota and liver metabolites

**Fig. 6:**
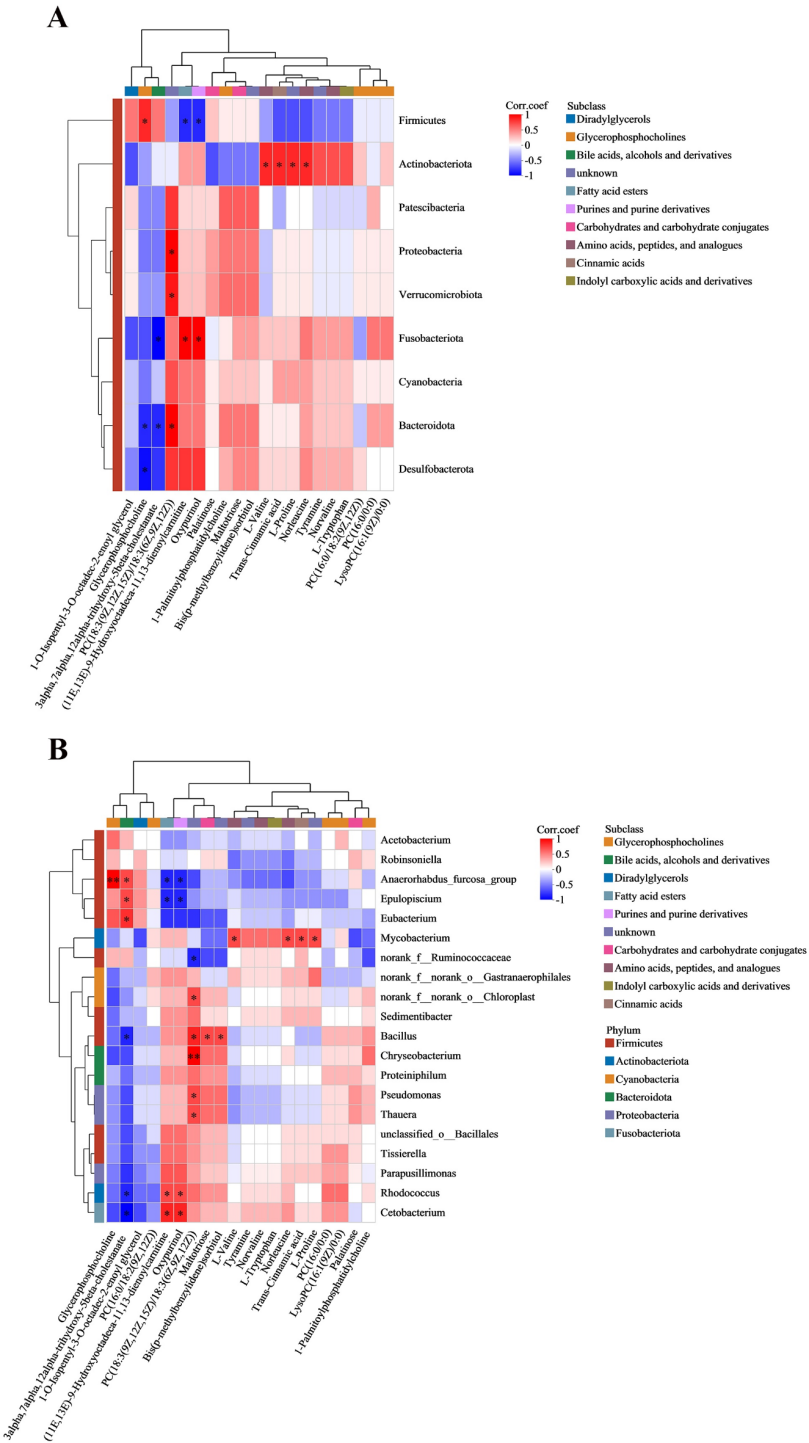
Correlation between liver metabolite and gut microorganism in CT10 VS CON. (A) Heatmap of the association between liver metabolite and gut microorganism on the portal category. (B) Heatmap of the association between liver metabolites and gut microbes on genus categories. * indicates statistically significant differences (* *P* < 0.05, ** *P* < 0.01). Each block grid represents the correlation between metabolite and microorganism, with different colours indicating the magnitude of the correlation coefficient. PC(18:3(9Z,12Z,15Z)/18:3(6Z,9Z,12Z)) (Phosphatidylcholine).

The heat map of Spearman’s correlation results was used to evaluate the correlation between intestinal microbiota and liver metabolites after treatment with the environmental level of concentration (10 µg/L) of chlorothalonil. For example, at the phylum level, the abundance of *Firmicutes* had a negative correlation with the abundance of oxypurinol and (11E,13E)-9-hydroxyoctadeca-11,13-dienoylcarnitine metabolites, and it had a positive correlation with the abundance of glycerophosphocholine. The abundance of *Actinobacteriota* had a positive correlation with the abundance of L-proline, L-valine, trans-cinnamic acid and norleucine (Fig. 6A). At the genus level, the abundance of *Mycobacterium* had a positive correlation with the abundance of L-proline, L-valine, trans-cinnamic acid and norleucine. The abundance of *Pseudomonas* had a positive correlation with the abundance of phosphatidylcholine (PC). The abundance of *Rhodococcus* had a positive correlation with the abundance of oxypurinol and (11E,13E)-9-Hydroxyoctadeca-11,13-dienoylcarnitine, while it had a significant negative correlation with 3α,7α,12α-trihydroxy-5β-cholestanate (Fig. 6B).

## Discussion

### Disruption of locomotor behavior by chlorothalonil in *P. nigromaculatus* tadpoles

Locomotion is an important indicator used in toxicology to assess the toxicity of contaminants^4^. In this study, chlorothalonil treatment significantly reduced locomotion speed and locomotion frequency in *P. nigromaculatus* tadpoles. Other studies have also found that pesticides harmed animal locomotion ability^45^. For example, Ghose et al.^27^ found that exposure to chlorothalonil at 12.5 µg/L affects the activity of *Agalychnis callidryas* larvae, while 31.2 mg/L ethoprophos can affected activity of tadpole^27^. Furthermore, chlorothalonil treatment causes behavioral changes in *Rhinella arenarum* larvae, such as erratic/circular swimming and weakness^28^.

### Effects of chlorothalonil exposure on intestinal microbiota of *P. nigromaculatus* tadpoles

Intestinal microbiota is a major contributor to host metabolism and plays an important role in regulating important processes such inflammation and immunity^46^. We observed significant differences in α-diversity and β-diversity between the control groups and exposure groups at both concentrations. Chlorothalonil exposure also reduced the community structure and evenness of gut microorganisms in *P. nigromaculatus* tadpoles, similar to the results observed by Huang et al.^47^. Among the various indices we considered, the Inverse-Simpson index, the Pielou index, and the Shannon’s evenness index were significantly reduced in CT10 and CT50 *P. nigromaculatus* tadpoles. Previous studies have found that fungicides such as chlorothalonil, pyraclostrobin and azoxystrobin can interfere with gut flora^12,48,49^. For example, chlorothalonil induces intestinal microbiota imbalance in mice, and is thought to be a potential endocrine disruptor^12^.

The present study confirmed that at the phylum level, *Firmicutes* and *Actinobacteriota* were the dominant phylum in *P. nigromaculatus* tadpoles, and chlorothalonil exposure significantly increased the abundance of *Firmicutes* and significantly decreased the abundance of *Actinobacteriota*. Previous studies have found that *Firmicutes* are the dominant microbiota in animal intestines, and an increase in *Firmicutes* abundance indicated an inflammatory bowel disease^50,51^. *Actinobacteriota* is a gram-positive bacteria that plays a key role in maintaining intestinal immunity, digestion, and absorption capacity^52–54^. The changes in these dominant bacterial phyla in the intestine after exposure to chlorothalonil indicated a significant disruption of the steady-state regulatory mechanisms.

At the genus level, chlorothalonil exposure significantly reduced the relative abundance of *Pseudomonas* and *Rhodococcus*. *Pseudomonas* is a probiotic that plays an important role in protecting aquatic animals from pathogens^55,56^. For example, *Pseudomonas* can stimulate the production of immunity to prevent rainbow trout (*Oncorhynchus mykiss*) fry syndrome, which in turn reduces the mortality rate of rainbow trout fry infected with *Flavobacterium psychrophilum*^57^. *Rhodococcus* benefits the metabolic processes of the host and can promote the absorption of nutrients from the host intestine^55,58^. Nutrients are a source of energy and play an important role in maintaining normal locomotion^10,59,60^. Interestingly, *Rhodococcus* has also been found to be significantly correlated with two performance metrics analyzed for locomotion, including movement time and movement distance^61^. Thus, exposure to chlorothalonil could lead to malnutrition in *P. nigromaculatus* tadpoles, with malnutrition being a possible factor in the altered locomotion of *P. nigromaculatus* tadpoles.

### Effects of chlorothalonil exposure on gut metabolic pathways and metabolites

The results of gut metabolomics in this study found that chlorothalonil exposure altered the metabolic profile of *P. nigromaculatus* tadpoles. This result has also been found by previous studies where continuous exposure to environmental contaminants could alter multiple metabolic pathways in *P. nigromaculatus* tadpoles^34^. Specifically, exposure to chlorothalonil significantly decreased the gut metabolites (e.g., phenylpyruvic acid and shikimic acid) and significant changed the amino acid-related pathways (e.g., tryptophan metabolism, phenylalanine, tyrosine and tryptophan biosynthesis, and alanine, aspartate and glutamate metabolism). We observed that phenylpyruvic acid and shikimic acid are metabolites of the phenylalanine, tyrosine and tryptophan biosynthesis pathway. Phenylpyruvic acid is not only associated with the immune system, but is also an intermediate in the metabolism of phenylalanine, which can be converted to phenylalanine among the essential amino acids^62,63^. Shikimic acid is a chiral carbon structure compound that acts as a key aromatic mediator in the synthesis of L-tyrosine, L-phenylalanine, and L-tryptophan. It ameliorates intestinal inflammation by inhibiting the production of inflammatory factors such as TNF-α, IL-1β, and IFN-γ, which in turn ameliorates intestinal inflammation^64^. Notably, damage to the GI tract disrupts nutrient absorption and energy acquisition^60^. Therefore, the significant decrease in the abundance of phenylpyruvic acid and shikimic acid after sustained exposure to chlorothalonil may pose a threat to the intestinal health of *P. nigromaculatus* tadpoles.

Chlorothalonil treatment also significantly reduced indole-3-acetamide levels and inhibited the tryptophan metabolism pathway in *P. nigromaculatus* tadpoles. It has been shown that gut microorganisms can directly or indirectly influence tryptophan metabolism and alter locomotion accordingly^65^. Tryptophan supports the development of the enteric nervous system, and its dysregulation plays a central role in the pathogenesis of many neurological and psychiatric disorders^65^. Indole-3-acetamide is a product of tryptophan catabolism, and may not only be a potential marker of depression-like behaviors (such as immobility), but also correlates significantly with gut bacteria^66–68^. Thus, exposure to chlorothalonil may affect tryptophan metabolic pathways directly or indirectly by interfering with the intestinal microbiota of *P. nigromaculatus* tadpoles. However, the mechanism of influence between specific gut microorganisms and tryptophan metabolism still needs to be further investigated.

### Effects of chlorothalonil exposure on liver metabolic pathways and metabolites

Liver metabolomics analysis showed that the metabolomic profile of the exposed groups were significantly different from that of the control group. In the liver, chlorothalonil significantly affects the cysteine and methionine metabolism pathway. Cysteine and methionine imbalances are characteristic of liver disease^69,70^. Significantly lower levels of L-cystathionine, 2-aminoacrylate and L-aspartic 4-semialdehyde metabolites in the livers of *P. nigromaculatus* tadpoles indicate that this metabolic process is interrupted. Furthermore, it has been shown that the hepatotoxicity of cantharidin (CTD) is associated with an increase in lipid peroxidation, as the L-cystathionine of the cysteine and methionine metabolism pathway is not only significantly reduced, but L-cystathionine could reduce lipid peroxidation^71^. These results indicate that chlorothalonil harmed the liver of *P. nigromaculatus* tadpoles.

This study also observed that different concentrations of chlorothalonil exposure had different effects on liver metabolites. For example, phosphatidylcholine (PC) abundance of the glycerophospholipid metabolism pathway was significantly reduced in CT10 compared to CON. Phosphatidylcholine (PC) plays an important role in protecting the liver^72,73^. In addition, the levodopa content of the tyrosine metabolism pathway was significantly lower in CT50 compared to CON. Levodopa has been shown to have efficacy in improving locomotion disorders^74^. It has also been reported to alleviate Carboplatin-induced hepatotoxicity^75^. The liver injury of *P. nigromaculatus* tadpoles in CT10 and CT50 may have been caused by significantly lower levels of Phosphatidylcholine and Levodopa. Levodopa is a potential key metabolite causing abnormal locomotion in *P. nigromaculatus* tadpoles, although Levodopa was not significantly reduced in CT10.

### Effects of exposure to environmental concentrations (10 µg/L) of chlorothalonil on the gut-liver axis of *P. nigromaculatus* tadpoles

The gut-liver axis as a new perspective in toxicological studies holds great promise for a deeper understanding of the toxic effects and mechanisms of pollutants^17^. Metabolites in the liver (such as bile acids) not only enter the gut via the bile ducts and systemic circulation, but also exert tight control over the intestinal microbiota, effectively maintaining the homeostasis of the gut-liver axis^76–78^. In this study, a significant increase in glycerophosphocholine metabolites in the liver after treatment with environmental concentrations of chlorothalonil was significantly correlated with *Firmicutes*, which dominate the gut flora (Table S1). Glycerophosphocholine is a naturally occurring choline, and increasing the relative amount of choline in sows can lead to a significant increase in the relative abundance of the dominant phylum *Firmicutes*, similar to our findings^79^. It has been shown that significantly elevated levels of glycerophosphocholine are characteristic of liver lesions^80^.

Gut microorganisms and their metabolites can enter the liver through several pathways, affecting liver function and even the normal activities of the organism^78^. This study documented that the effect of chlorothalonil on intestinal microbiota could further lead to changes in the relative levels of liver metabolites. Oxypurinol had a significant decrease in relative abundance in the presence of environmental concentrations of chlorothalonil (Table S1) and a significant positive association with the beneficial gut bacterial genus *Rhodococcus*, which may indicate that a decrease in the abundance of *Rhodococcus* leads to a decrease in the levels of oxypurinol metabolites. Oxypurinol, the major metabolite of allopurinol, has been shown to inhibit hepatocyte damage^81,82^. Furthermore, intestinal microbiota may alter different liver metabolites by affecting these pathways. In our work, the relative abundance of the phospholipid-related metabolite phosphatidylcholine in the hepatic glycerophospholipid metabolism pathway, as well as of intestinal *Pseudomonas*, was significantly reduced by the treatment of ambient concentrations of chlorothalonil (Table S1). Wang et al.^83^ reported that the phospholipid-related metabolite PC (20:1/22:6) was significantly positively correlated with *Pseudomonas*, which is consistent with the trend of having a significant positive correlation between PC (18:3(9Z,12Z,15Z)/18:3(6Z,9Z,12Z)) (phosphatidylcholine) and *Pseudomonas*. It has been shown that *Pseudomonas plecoglossicida*, a member of *Pseudomonas*, can cause hepatotoxicity^84^. Although we have no direct evidence that *Pseudomonas* can lead to the outcome of disturbed glycerophospholipid metabolism, hepatotoxicity implies the alteration of phospholipid-related metabolites in the glycerophospholipid metabolic pathway^85^. Interestingly, the process of phospholipid synthesis is widely known to be involved in energy metabolism and the synthesis of essential substances, which are important for the locomotion ability of an organism^86^.

**Fig. 7:**
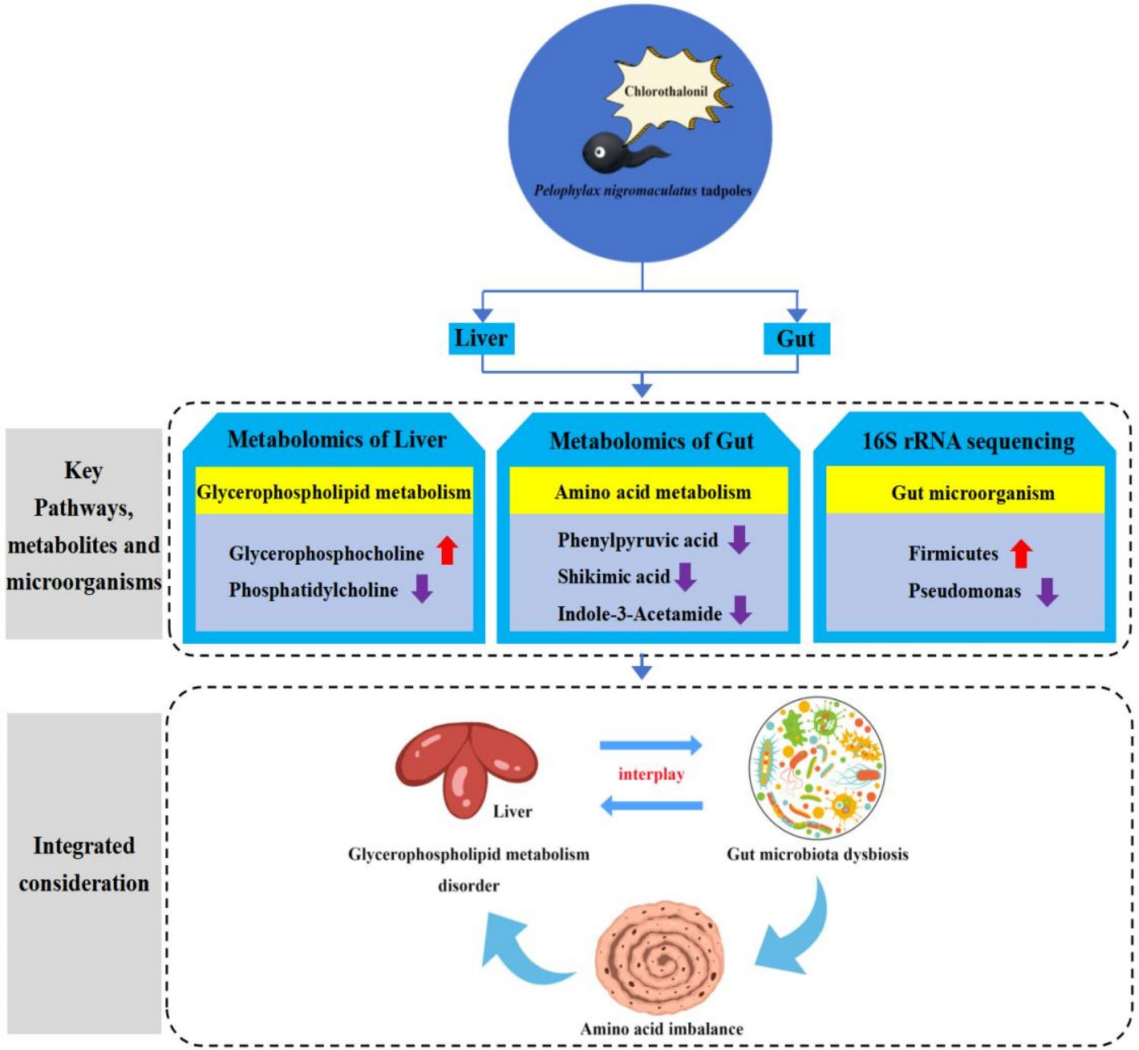
Potential mechanisms by which environmental concentrations (10 µg/L) of chlorothalonil treatments cause alterations in the gut-liver axis homeostasis in *P. nigromaculatus* tadpoles. The main mechanisms included: (1) Exposure to environmental concentrations of chlorothalonil can lead to disturbances in the intestinal microbiota and liver damage, and liver interacts with intestinal microbiota. (2) Gut dysbiosis induced by exposure to environmental concentrations of chlorothalonil mediates amino acid imbalances in the gut, which in turn disrupts glycerophospholipid metabolism in the liver. Dashed arrows indicate possible causes. Red and purple arrows indicate significant increases and significant decreases respectively.

In addition, intestinal microbiota performs complex metabolic activities to provide the energy and nutrients they need to grow and reproduce, as well as produce a large amount of metabolites in the host. Altered or ecologically dysregulated gut flora may lead to metabolic abnormalities^87^. Gut metabolomics has shown that pathways related to amino acid metabolism in the gut are significantly affected following exposure to environmental concentrations of chlorothalonil, which is associated with the development of inflammation^88^. Amino acid metabolism plays a crucial role in the intestinal microbiota, and its disruption may affect the energy supply in *P. nigromaculatus* tadpoles after chlorothalonil exposure^89^. Furthermore, *Firmicutes* are responsible for the metabolism of aromatic amino acids^90^. In the present study, chlorothalonil-induced enrichment of *Firmicutes* may be associated with amino acid-related metabolic disorders in the gut of *P. nigromaculatus* tadpoles. It has been reported that most of the amino acids produced in the gut after cyclophosphamide exposure cause liver injury via the gut-liver axis^91^. Amino acid imbalances in the gut are a potential factor in disorders of glycerophospholipid metabolism, as liver injury is characterized by disorders of glycerophospholipid metabolism. In summary, environmental concentrations of chlorothalonil treatments may cause alterations in the gut-liver axis homeostasis in *P. nigromaculatus* tadpoles (Fig. 7).

## Conclusion

Chlorothalonil is a broad-spectrum, non-systemic fungicide that affects the aquatic environment. This experiment investigated the behavioral changes induced by chlorothalonil exposure from the perspective of behavior analysis, and this experiment investigated the mechanisms of alterations the homeostasis of gut-liver axis induced by chlorothalonil exposure from the perspective of microbial diversity analysis and metabolomics. Behavioral analyses confirmed that chlorothalonil exposure inhibited the locomotor ability of *P. nigromaculatus* tadpoles, which had a significant reduction in mean speed and frequency of locomotion. The results of gut microbial diversity analysis, gut metabolomics and liver metabolomics suggested that chlorothalonil exposure affected gut microorganisms, gut metabolism and liver metabolism, and that these changes posed a threat to the health of *P. nigromaculatus* tadpoles. Comprehensive analyses of the gut-liver axis suggested that environmental concentrations of chlorothalonil could cause alterations in the homeostasis of the gut-liver axis.

## Electronic supplementary material

Below is the link to the electronic supplementary material.

Supplementary Material 1

## Data Availability

The data presented in the study are deposited in the NCBI repository with the identifier PRJNA1220183. Available at: https://dataview.ncbi.nlm.nih.gov/object/PRJNA1220183?reviewer=askcqaa7iimvo0523tdpjg4svv. The additional data generated in the current study are available from the corresponding author on reasonable request.
