# Supplementary material for: Chronic chlorothalonil exposure inhibits locomotion and interferes with the gut-liver axis in Pelophylax nigromaculatus tadpoles
Source: Sci Rep. 2025 Apr 25;15:14573. doi: 10.1038/s41598-025-98081-1 (PMC12032272; doi:10.1038/s41598-025-98081-1)
Supplement: Supplementary file 1 — Supplementary Material 1 [file 41598_2025_98081_MOESM1_ESM.docx]

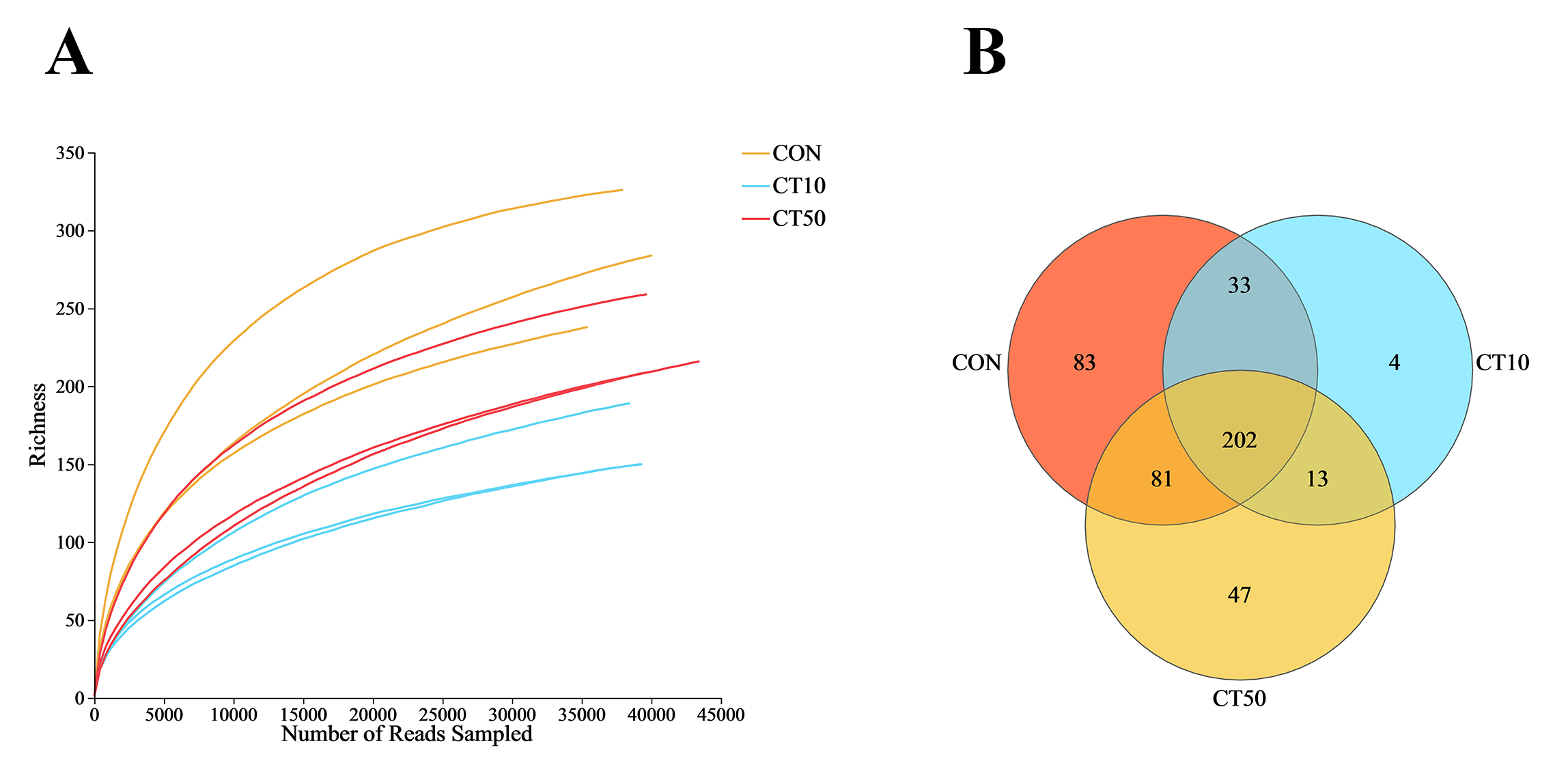


**Fig. S1** Dilution curves (A) and Venn analyses (B) of gut microorganism in CON, CT10 group and CT50 group in *P. nigromaculatus* tadpoles.


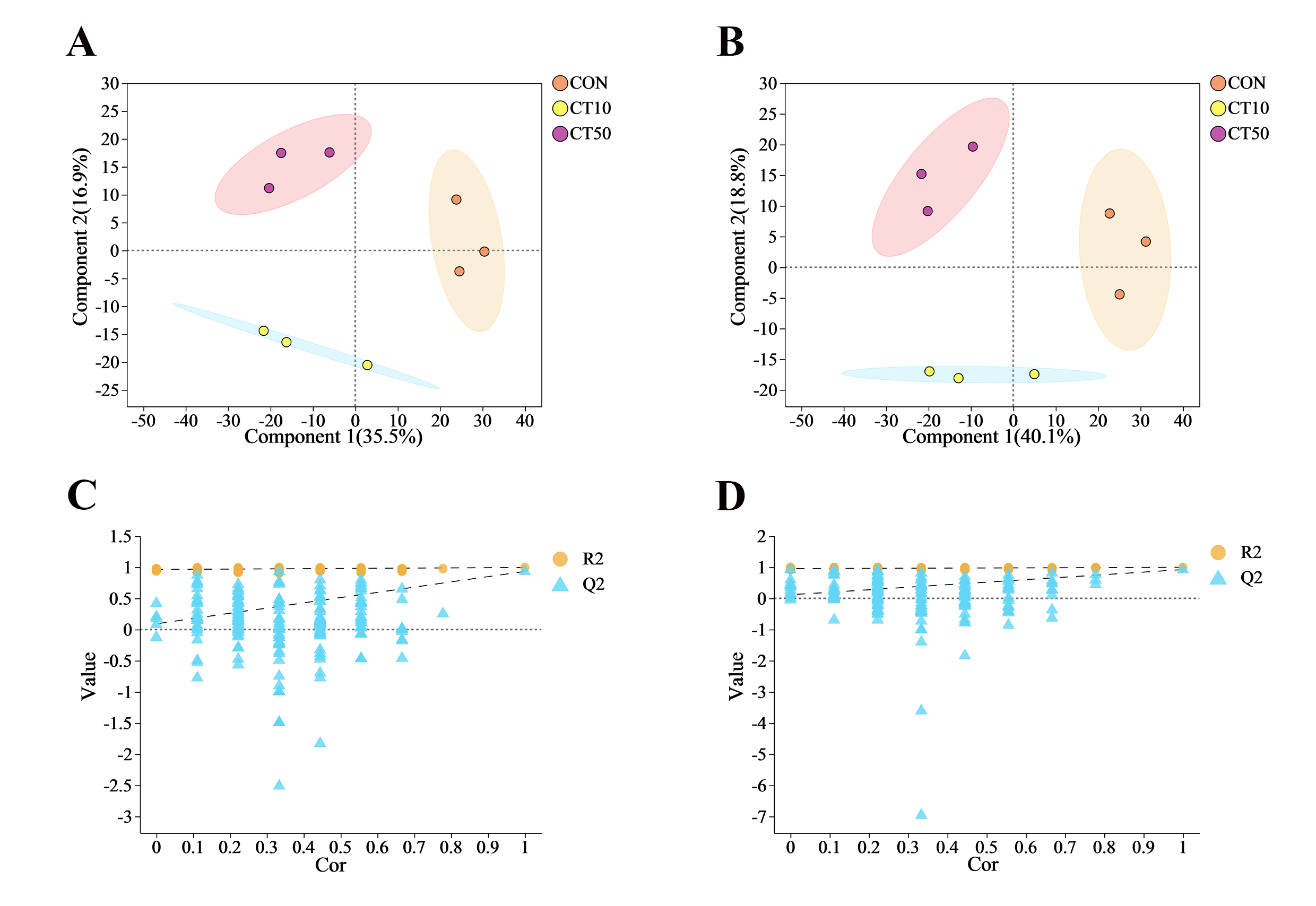


**Fig. S2** Non-targeted metabolomics analysis of liver of *P. nigromaculatus* tadpoles. PLS-DA score plots from the CON, CT10 and CT50 in (A) positive mode and (B) negative mode. (C) and (D) PLS-DA model validation. Validation plots were obtained in (A) positive mode (R2 = 0.964, Q2 = 0.0929) and (B) negative mode (R2 = 0.9558, Q2 = 0.1163).

| Liver metabolites | Metabolic pathway in which the metabolite is located | CON  (mean ± SD) | CT10  (mean ± SD) |
| --- | --- | --- | --- |
| PC(18:3(9Z,12Z,15Z)/18:3(6Z,9Z,12Z)) | Glycerophospholipid  metabolism | 7.9531 ± 0.01557 a | 7.6731 ± 0.13889 b |
| Oxypurinol | - | 7.7767 ± 0.01495 a | 7.6527 ± 0.01032 b |
| Glycerophosphocholine | Glycerophospholipid metabolism | 7.9513 ± 0.15755 a | 8.3018 ± 0.02886 b |

**Table S1. Important differential metabolites in CON and CT10 group**
